# Increased lncRNA ABHD11-AS1 represses the malignant phenotypes of bladder cancer

**DOI:** 10.18632/oncotarget.14945

**Published:** 2017-02-01

**Authors:** Mingwei Chen, Jianfa Li, Chengle Zhuang, Zhiming Cai

**Affiliations:** ^1^ Department of Urology, The Fourth Affiliated Hospital of Zhejiang University School of Medicine, Yiwu 322000, Zhejiang, China; ^2^ Shantou University Medical College, Shantou 515041, Guangdong, China; ^3^ Peking University Shenzhen Hospita, Shenzhen 518036, Guangdong, China

**Keywords:** ABHD11-AS1, bladder cancer, lncRNAs, proliferation, migration

## Abstract

Bladder cancer is one of the most common urothelial tumors worldwide. While there are some progresses on early bladder cancer detection, patients’ mortalities have not been changed significantly. So it is important to get further understanding the mechanism involved in the development and progression of bladder cancer. Long non-coding RNAs play important regulatory roles in a variety of biological processes ranging from gene regulation, cellular differentiation to tumorigenesis. Previous literatures reported that lncRNA ABHD11 Antisense RNA 1 (ABHD11-AS1) (Organism: Homo sapiens) was highly expressed in gastric cancer. Inspired by these observations, we hypothesized that ABHD11-AS1 possibly plays an analogous role in human bladder cancer. We first found that ABHD11-AS1 was up-regulated in bladder cancer tissues and cell lines, and ABHD11-AS1 expression level was positively associated with clinicobiological features. Cell proliferation, cell migration and apoptosis were observed by silencing ABHD11-AS1 and overexpression ABHD11-AS1 caused contrary effects. Taken together, these data suggested that ABHD11-AS1 may be an oncogene and a therapeutic target in bladder cancer.

## INTRODUCTION

Malignant bladder carcinomas are the most common tumors in urinary systems. Although the diagnosis and treatment of bladder cancer has been much improved during recent years [1–5], there are still many questions to be solved. Moreover, the ratios of recurrence, metastasis and death still remain high, which cause heavy burden to the society [6]. Therefore, in order to explore new therapeutic targets for bladder cancer patients who are not sensitive to current treatments, further studies on bladder cancer development are needed [7, 8]. Long non-coding RNAs (lncRNAs) belong to a kind of non-coding RNAs which had been regarded as useless molecules in the past. They do not contain open-reading frames, but have conservative secondary structures. LncRNAs interacted with DNA, RNA or proteins as molecular sponges, scaffolds and activators to play important regulatory roles in a variety of biological processes. We supposed that ABHD11-AS1 should affect apoptosis through a similar way. LncRNAs mainly regulated and controlled gene transcription, cell differentiation, epigenetic and other life activities [9–23]. For example, PVT1 was reported to be highly expressed and to control cellular behaviors in bladder cancer by our group [24]. In summary, lncRNAs function as oncogenes or tumor suppressors in human tumor diseases, which promote cancer or suppress tumors [25–38]. But many studies on lncRNAs were still in the primary stage, there's a long way to transform them into application in clinical diagnosis and treatment.

Alpha/beta hydrolase domain (ABHD)-containing proteins are structurally related with diverse catalytic activities. In various species, some ABHD proteins have been characterized and shown to play roles in lipid homeostasis. AT4G10030AtABHD11 is a lyso (phospho) lipase. The disruption of AtABHD11 caused the accumulation of the polar lipids in leaves, which in turn promoted a higher growth rate compared to wild-type plants [39, 40]. ABHD11 and Esterase D can be used to predict the development of distant metastases of aggressive lung adenocarcinomas [41]. The abnormally upregulation of ABHD11 Antisense RNA 1 (ABHD11-AS1) (Organism: Homo sapiens) has important clinical significances in gastric cancer [42, 43]. Abhd11os(Organism:Mus musculus, called ABHD11-AS1 in human) may play crucial roles in neurodegenerative diseases [44]. These studies suggested that the lncRNA ABHD11-AS1 perhaps involved in other diseases, such as bladder cancer.

In this study, we first reported that long non-coding RNA ABHD11-AS1 was up-regulated in the bladder cancer tissues and cell lines. We explored the correlation between expression level of long non-coding RNA ABHD11-AS1 and the clinicopathologic features. In this research, we also investigated the effects of ABHD11-AS1 on cell proliferation, cell migration, and cell apoptosis in bladder cancer through inhibition or over-expression of ABHD11-AS1. Our findings provide new insights into the role of the lncRNA ABHD11-AS1 in the bladder cancer.

## RESULTS

### Up-regulation of ABHD11-AS1 in bladder cancer and its correlation with clinical pathologic factors

**Figure 1:**
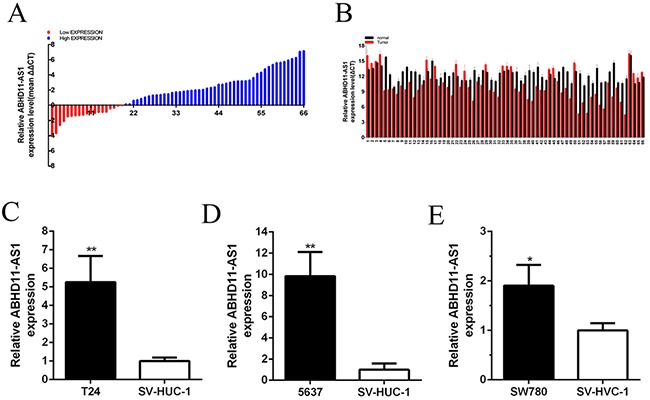
ABHD11-AS1 was overexpressed in bladder cancer tissues and cell lines **A**. qPCR was utilized to detect relative concentration of ABHD11-AS1 in bladder cancer. **B**. The relative expression level of ABHD11-AS1 was increased in bladder cancer tissues compared with normal tissues by using paired discrete plots. Compared to SV-HUC-1, the relative expression level of ABHD11-AS1 was increased in bladder cancer T24 **C**. 5637 **D**. and SW780 **E**. cells. The data of each group is multiplied by (−1) in Figure 1. Assays were performed in triplicate. All data are shown as mean ± SD (*p < 0.05, **p < 0.01).

The expression level of ABHD11-AS1 was examined by carrying out quantitative real-time PCR in bladder cancer tissues and pair-matched adjacent normal bladder tissues from 66 bladder cancer patients (Figure 1A). The ABHD11-AS1 expression was up-regulated in 71.2% (47 of 66) of cancer tissues. Compared with pair-matched adjacent normal bladder tissues, the ABHD11-AS1 expression was up-regulated significantly in bladder cancer tissues (*p*<0.01 by Paired samples *t* test) (Figure 1A and 1B). As shown in Table 1, upregulation of ABHD11-AS1 was highly correlated with bladder cancer clinical pathologic grading (*p*<0.001) and tumor invasion depth (p=0.001). TNM stage also had a high association with up-regulated expression of ABHD11-AS1 (*P*=0.001). But gender, age, tumor invasion depth and lymph node metastasis had no associations with ABHD11-AS1 expression level. Assays were performed in triplicate.

**Table 1:** Correlation between ABHD11-AS1 expression and clinicopathological characteristics of bladder cancer patients

Characteristics	Expression of ABHD11-AS1		P value
	High (n=47)	Low (n=19)	
Gender			
Male	33(73.3%)	12(26.7%)	0.575
Female	14 (66.7%)	7(33.3%)	
Age			
<=60	25(75.8%)	8(24.2%)	0.587
>60	22(66.7%)	11(33.3%)	
Histological grade			
PUNLMP/Low-grade	10(41.7%)	14(58.3%)	P<0.001**
High-grade	37(88.1%)	5(11.9%)	
Tumor invasion depth (T)			
Tis, Ta, T1	15(50.0%)	15(50.0%)	0.001**
T2, T3 or above	32(88.9%)	4(11.1%)	
Lymph node metastasis(N)			
N0	41(69.5%)	18(30.5%)	0.663
N1 or above	6(85.7%)	1(14.3%)	
TNM stage			
0/I	12(46.2%)	14(53.8%)	0.001**
II/III/IV	35(87.5%)	5(12.5%)	

PUNLMP, papillary urothelial neoplasm of low malignant potential; Low-grade, lowgrade papillary urothelial carcinoma; High-grade, high-grade papillary urothelial carcinoma (http://www.pathology.jhu.edu/bladder).

TNM according to the seventh edition of staging TNM of Union Internationale Contre Le Cancer (UICC) in 2009.

** P < 0.01

### Up-regulation of ABHD11-AS1 in bladder cancer cell lines

Quantitative real-time PCR was used to measure the expression level of ABHD11-AS1 in bladder cancer cell lines. Compared with the SV-HUC-1, the ABHD11-AS1 expression was increased in bladder cancer cell lines 5637 (P = 0.000 294), T24 (P = 0.000 963) and SW780 (P = 0.039 956) (Figure 1C, 1D and 1E). Assays were performed in triplicate.

### ABHD11-AS1 promoted cell proliferation

We further determined whether ABHD11-AS1 promoted cell proliferation in bladder cancer. The relative expression levels of ABHD11-AS1 were analyzed by qRT-PCR at 48 hours after transfection of siRNAs or plasmids in bladder cancer cell lines.

**Figure 2:**
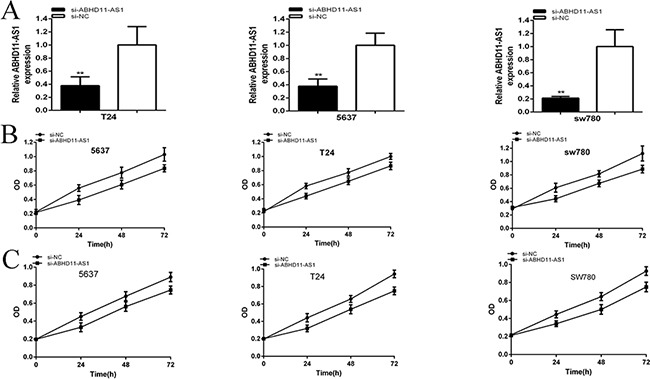
qRT-PCR was used to measure the changes in expression levels of ABHD11-AS1 after transfection of specific RNA **A**. CCK8 was used to detect cell proliferation after transfection of specific RNA **B**. MTT was used to detect cell proliferation after transfection of specific RNA **C**. Assays were performed in triplicate. Data are illustrated as mean ± SD (**p < 0.01).

The relative expression levels of ABHD11-AS1 in 5637(p=0.007576), T24 (P=0.006413) and SW780 (p=0.000918) cells were down-regulated significantly by siRNA at 48 hours post transfection (Figure 2A). In the 5637 cell lines, the relative expression of ABHD11-AS1 was decreased by 62.30%. In T24 cell lines, the relative expression was decreased by 62.31%. In SW780 cell lines, the relative expression was decreased by 78.66%. CCK-8 and MTT assays demonstrated that si-ABHD11-AS1 inhibited cell proliferation remarkably in bladder cancer cells (p < 0.001 in three cell lines) (Figure 2B and 2C).

**Figure 3:**
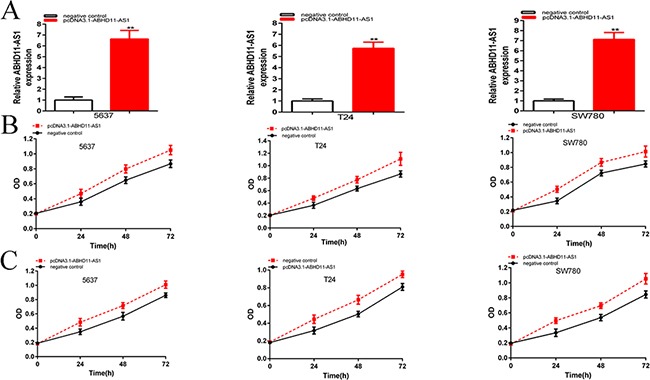
qRT-PCR was used to measure the changes in expression levels of ABHD11-AS1 after transfection of specific plasmids **A**. CCK8 was used to detect cell proliferation after transfection of specific plasmids **B**. MTT was used to detect cell proliferation after transfection of plasmids **C**. Assays were performed in triplicate. Data are illustrated as mean ± SD (**p < 0.01).

And the relative expression levels of ABHD11-AS1 were up-regulated significantly at 48 hours post transfection of pcDNA3.1-ABHD11-AS1 in 5637(p=0.00031), T24 (P=0.000151) and SW780 (p=0.000115) cells (Figure 3A). In the 5637 cell lines, the relative expression of ABHD11-AS1 was up-regulated about 6.64-fold over the control. In T24 cell lines, the relative expression of ABHD11-AS1 was up-regulated about 5.74-fold over the control. In SW780 cell lines, the relative expression of ABHD11-AS1 was up-regulated about 7.13-fold over the control. CCK-8 and MTT assays showed that pcDNA3.1-ABHD11-AS1 increased cell proliferation remarkably in bladder cancer cells (p < 0.001 in three cell lines) (Figure 3B and 3C).

**Figure 4:**
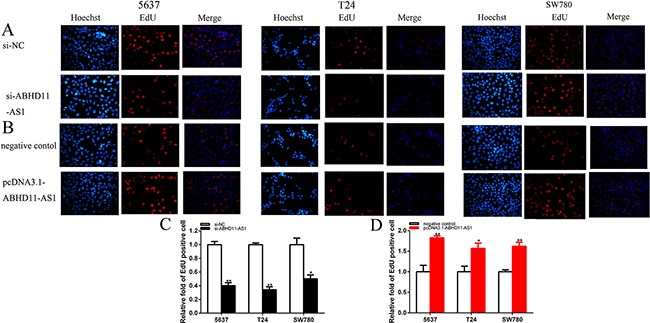
EDU was used to detect cell proliferation after transfection specific RNA **A**. and **C**. and specific plasmids **B**. and **D**. Assays were performed in triplicate. Data are illustrated as mean ± SD (*p < 0.05, **p < 0.01).

EdU assay was further illustrated to detect cell proliferation. As shown in Figure 4A–4D, compared to si-NC, EdU positive 5637, T24 and SW780 cells in si-ABHD11-AS1 were reduced after transfection, and compared to negative control group, EdU positive 5637, T24 and SW780 cells in pcDNA3.1-ABHD11-AS1 group were increased after transfection. EdU assay illustrated that the quantity of EdU positive cells in si- ABHD11-AS1 group was reduced by 59.9% in 5637 (P = 0.005 520), decreased by 65.9% in T24 (P = 0.0028 849) and suppressed by 49.8%in SW780 (P = 0.024 164) (Figure 4A and 4C). The quantity of EdU positive cells in pcDNA3.1-ABHD11-AS1 group was increased by 1.83-fold in 5637 (P= 0.006 370), increased by 1.57-fold in T24 (P= 0.018 482) and increased by 1.62-fold in SW780 (P = 0.002 179) (Figure 4B and 4D).

The results demonstrated that ABHD11-AS1 promoted cell proliferation remarkably in bladder cancer. Assays were performed in triplicate.

### ABHD11-AS1 accelerated cell migration

**Figure 5:**
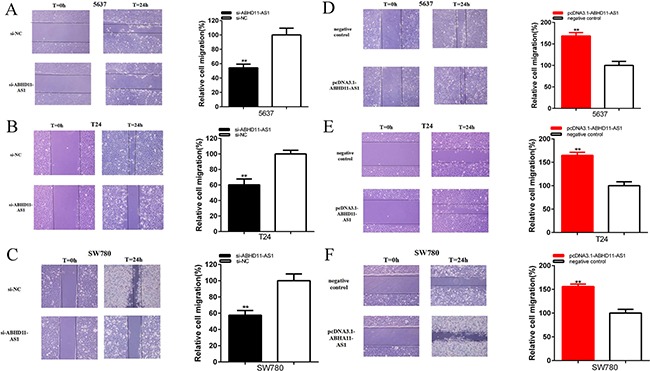
Effects of specific RNA and specific plasmids on cell migration Cell migration was suppressed in si-ABHD11-AS1 group compared with si-NC group in 5637 **A**. T24 **B**. and SW780 **C**. Cell migration was suppressed in pcDNA3.1-ABHD11-AS1 group compared with negative control group in 5637 **D**. T24 **E**. and SW780 **F**. Assays were performed in triplicate. All data are shown as mean ± SD (*p < 0.05, **p < 0.01).

Cells were transfected with si-ABHD11-AS1 or pcDNA3.1- ABHD11-AS1 in 6-well plates. The cell scratch assay was utilized to detect the role of specific siRNA and plasmid in cell migration. Compared with si-NC group, cell migration of bladder cancer cells was significantly suppressed by si-ABHD11-AS1. Sratch assay illustrated that the ratio of the relative migration in is- ABHD11-AS1 group was reduced by 45.80% in 5637 (P = 0.001 821), decreased by 39.74% in T24 (P = 0.001 498) and lessen by 42.41% in SW780 (P = 0.001 955) (Figure 5A–5C).

Compared with negative control group, cell migration was also promoted by pcDNA3.1-ABHD11-AS1. Sratch assay illustrated that the ratios of the relative migration in PcDNA3.1- ABHD11-AS1 group were increased by 68.54% in 5637(P =0.000 641), increased by 64.93% in T24 (P = 0.000 444), increased by 55.93% in SW780 (p=0.000 492) (Figure 5D–5F).

These data suggested that ABHD11-AS1 increased cell migration in bladder cancer cells. Assays were performed in triplicate.

### ABHD11-AS1 suppressed apoptosis of bladder cancer cells

To investigate the effect of si-ABHD11-AS1 and pcDNA3.1-ABHD11-AS1 on the apoptosis of bladder cancer cells, the caspase-3 enzyme-linked immunesorbent assay (ELISA), Hoechst 33258 staining assay and flow cytometry were used to measure the apoptosis rate. Bladder cancer cells were transfected with siRNA and plasmid.

**Figure 6:**
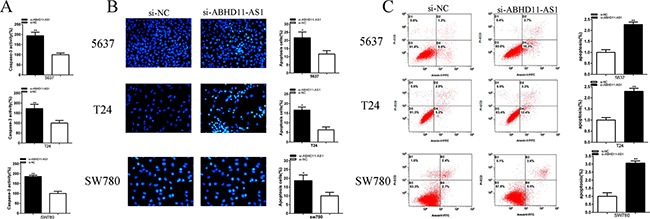
Effects of si-ABHD11-AS1 on apoptosis Apoptosis was detected by caspase 3 ELISA assays, Hoechst 33258 assays and flow cytometry assays. The activity of caspase-3 was higher significantly after transfection with si-ABHD11-AS1 in 5637, T24 and SW780 **A**. More apoptosis cells were measured significantly after transfection with si-ABHD11-AS1 in 5637, T24 and SW780 **B**. Apoptosis ratio was observed using flow cytometry assays. More apoptotic cells were detected in si-ABHD11-AS1 group compared with si-NC group in 5637, T24 and SW780 **C**. Assays were performed in triplicate. All data are shown as mean ± SD (*p < 0.05, **p < 0.01).

Compared with si-NC group, the activities of caspase-3 were significantly increased by si-ABHD11-AS1 (Figure 6A). ELISA assay illustrated that the activities of caspase-3 in si- ABHD11-AS1 group were increased by 94.25% in 5637 (P = 0.001 06), increased by 72.55% in T24 (P = 0.007 508) and increased by 85.91% in SW780 (P =0.000 306).

Compared with si-NC group, the number of apoptotic cells was significantly increased by si-ABHD11-AS1 (Figure 6B). Hoechst 33258 staining assay illustrated that the number of apoptotic cells in si- ABHD11-AS1 group was increased 1.86-fold in 5637 (P = 0.013 236), increased 2.63-fold in T24 (P = 0.011 159) and increased 1.87-fold in SW780 (P = 0.016 609).

Compared with si-NC group, the ratios of apoptosis were significantly increased by si-ABHD11-AS1 (Figure 6C). Flow cytometry assay illustrated that the ratios of apoptosis in si- ABHD11-AS1 group was increased 2.27-fold in 5637 (P = 0.008 649), increased 2.29-fold in T24 (P = 0.009 344) and increased 3.06-fold in SW780 (P = 0.007 715).

**Figure 7:**
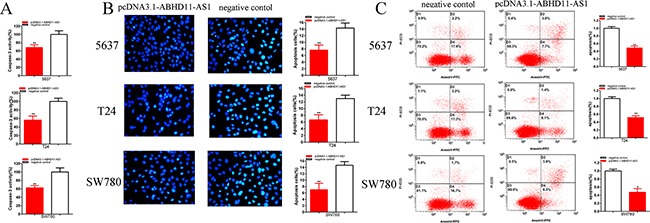
Effects of pcDNA3.1-ABHD11-AS1 on apoptosis Apoptosis was detected by caspase 3 ELISA assays, Hoechst 33258 assays and flow cytometry assays. The activity of caspase-3 was higher significantly after transfection with pcDNA3.1-ABHD11-AS1 5637, T24 and SW780 **A**. More apoptosis cells were measured significantly after transfection with pcDNA3.1-ABHD11-AS1 in 5637, T24 and SW780 **B**. Apoptosis ratio was observed using flow cytometry assays. More apoptotic cells were detected in pcDNA3.1-ABHD11-AS1 group compared with si-NC group in 5637, T24 and SW780 **C**. Assays were performed in triplicate. All data are shown as mean ± SD (*p < 0.05, **p < 0.01).

Compared with negative control group, the activities of caspase-3 were significantly decreased by pcDNA3.1-ABHD11-AS1 (Figure 7A). ELISA assay illustrated that the activities of caspase-3 in pcDNA3.1-ABHD11-AS1 group were decreased by 31.75% in 5637 (P = 0.008 301), decreased by 43.78% in T24 (P =0.003 404) and decreased by 37.42% in SW780 (P = 0.004 309).

Compared with negative control group, the number of apoptotic cells was significantly decreased by pcDNA3.1-ABHD11-AS1 (Figure 7B). Hoechst 33258 staining assay illustrated that the numbers of apoptotic cells in pcDNA3.1-ABHD11-AS1 group were decreased 1.87-fold in 5637 (P = 0.005 905), decreased 1.95-fold in T24 (P = 0.003 863) and decreased 2.10-fold in SW780 (P =0.004 535).

Compared with negative control group, the ratios of apoptosis were significantly decreased by pcDNA3.1-ABHD11-AS1 (Figure 7C). Flow cytometry assay illustrated that the ratios of apoptosis in pcDNA3.1-ABHD11-AS1group were decreased by 51.31% in 5637 (P = 0.008 542), decreased by 48.36% in T24 (P = 0.009 096) and decreased by 52.78% in SW780 (P = 0.018 003).

These results indicate that ABHD11-AS1 may reduce apoptosis in bladder cancer cells. Assays were performed in triplicate.

## DISCUSSION

Intermittent painless gross hematuria is one of the most common clinical symptoms in bladder cancer. Bladder malignant tumors could be divided into urothelial carcinoma, adenocarcinoma, squamous cell carcinoma, anaplastic carcinoma and other types in bladder tumors. The optimal surgical treatment mainly based on clinical pathology and clinical stage of bladder tumors. The transurethral resection of bladder tumor (TURBT) supplemented by postoperative bladder perfusion chemotherapy is the best therapeutic regimen for the non-muscle invasive bladder cancers (NMIBC), but easily relapsed. Radical cystectomy is the major treatment for the muscle invasive bladder cancers, but the life quality was still poor [1–4]. Therefore, new molecular markers [5–8] are needed to improve the diagnosis of bladder cancer.

Non-coding RNAs research started relatively late, and was regarded as no function and ignored transcription “noise”. Lots of literatures have illustrated that various miRNAs play significant roles in the bladder cancer progression. In recent years, accumulating evidences have shown that another type of non-coding RNAs –lncRNAs- represent an emerging player in cancer. In the past decade, many studies have shown that abnormal lncRNAs expression were closely related to diseases, especially tumors, and act as oncogenes or tumor suppressors to involve in the regulation of the tumor disease's growth and progression [30–38]. However, just a small proportion of lncRNAs have been illustrated in tumors. The dysregulation of ABHD11-AS1 in gastric cancer or nerve system have important impact on cells [42–44]. And we further studied the expression pattern and biological function of ABHD11-AS1 in bladder cancer.

This is the first report about the functions of ABHD11-AS1 in the development and progression of bladder cancer. In this study, expression level of ABHD11-AS1 in bladder cancer is highly related to its pathologic grade and clinical stage. Compared with the matched normal bladder cancer tissues, the expression of ABHD11-AS1 was up-regulated in bladder cancer tissues. Compared with SV-HUC-1, the expression of ABHD11-AS1 was up-regulated in bladder cancer cell lines. These data showed that the expression of ABHD11-AS1 was up-regulated in bladder cancer and cell lines and may represent as a new player in bladder cancer. To investigate the ABHD11- AS1 biological functions in bladder cancer, we used CCK8 assays, MTT assays, scratch assays and apoptosis assays to detect cell proliferation, cell migration and cell apoptosis, respectively. The si-ABHD11-AS1 suppressed cell growth and migration and induced cell apoptosis, and these effects could be reversed by pcDNA3.1-ABHD11-AS1. These results suggest that ABHD11-AS1 may play key roles in the malignant bladder cancer phenotype regulation.

In our study, this research elucidated that ABHD11-AS expression level is largely correlated with bladder cancer clinical pathologic grade and TNM phase. ABHD11-AS1 played oncogenic roles in the bladder cancer development and progression. ABHD11-AS1 may be a candidate biomarker for bladder cancer diagnosis and treatment, which could possibly change the present dilemma of the advanced bladder cancer treatment. But more efforts will be needed to find out the molecular mechanisms of ABHD11-AS1 in bladder cancer.

## MATERIALS AND METHODS

### Cell lines and cell culture

Three bladder cancer cell lines (T24, 5637, SW780) and a normal human uroepithelial cell line (SV-HUC-1) were all purchased from the Institute of Cell Research, Chinese Academic of Sciences, Shanghai, China. They were cultured as suggested by American Type Culture Collection (ATCC, Manassas, VA).

### Patient samples

This research was approved by the Human Ethics Committee of Shenzhen Second People's Hospital (Shenzhen, China) and The Fourth Affiliated Hospital of Zhejiang University School of Medicine (Yiwu, China). Written formal approval was also obtained from all the patients. Forty-sixth patients diagnosed with urothelial cancer by histopathological evaluation in the bladder were included in this study. Before biopsy, no any treatments were given to patients. Sixty-six bladder cancer tissues and matched non-tumor bladder tissues were acquired from patients and then snap-frozen in liquid nitrogen quickly.

### Quantitative real-time PCR

Total RNA from tissues or cells was obtained by using TRIzol reagent (Invitrogen, CA). RNA was reversed transcribed to cDNA by utilizing PrimeScript RT Reagent Kit with gDNA Eraser (Takara, Dalian, China). The primer sequences were shown: ABHD11-AS1 primers [42] forward: 5′–GAACGGGATGAAGCCATTG-3′, reverse: 5′-GCTGATTCTGGACCTGCTG-3′; GAPDH primers forward: 5′-CGCTCTCTGCTCCTCCTGTTC-3′ reverse: 5′–ATCCGTTGACTCCGACCTTCAC-3′. Quantitative PCR (qPCR) was executed using SYBR Green PCR kit (Takara, Dalian, China) following the manufacturer's instructions. GAPDH was measured as the internal control. The reactions were carried out on an ABI PRISM 7300 Fluorescent Quantitative PCR System (Applied Biosystems, Foster City, CA, USA) in triplicate. The 2^−ΔΔCt^ method was utilized to analyze the data and to calculate the relative quantitation of gene expression levels. Assays were performed in triplicate.

### Small interfering RNA (siRNA)

Specific siRNA oligonucleotides targeting ABHD11-AS1, ABHD11-AS1 siRNA (si-ABHD11-AS1), and negative control siRNA (si-NC) were synthesized by GenePharma, Suzhou, China. The sequences of ABHD11-AS1 siRNA were as follows: sense: GGGAUGAAGCCAUUGCUAATT and antisense: UUAGCAAUGG CUUCAUCCCTT. Bladder cancer cells were cultured in 6-well plates to get 30% confluence and then transfected with siRNAs using Lipofectamine 2000 (Invitrogen) according to the manufacturer's protocol.

### PcDNA3.1-ABHD11-AS1

The overexpressed vector, pcDNA3.1-ABHD11-AS1, was ordered from GenePharma (Suzhou, China). Negative Control was also purchased from GenePharma (Suzhou, China).

### CCK-8 assay

Cell proliferation was measured using Cell Counting Kit-8, CCK-8 (Beyotime Institute of Biotechnology, Shanghai, China) according to the manufacturer's instructions. 5×10^3^ cells per well were seeded in 96-well plates for 24 hours to get 30-50% confluence, and then transfected with siRNAs or plasmids. Assays were performed in triplicate.

### MTT assay

The proliferation of the bladder cancer cells was also determined by using 3-[4, 5-dimethylthiazol-2-yl]-2, 5-diphenyl-tetrazolium bromide (MTT) assay. Cells were grown in a 96-well plate for 24 hours, transfected with siRNAs or plasmids and cultured in normal medium. Cells were incubated in 0.1 mg/ml MTT for 4 hours and lysed in dimethyl sulfoxide (DMSO) at room temperature for 10 minutes. The absorbance in each well was calculated by using a microplate reader (Bio-Rad, Hercules, and CA). Assays were performed in triplicate.

### EDU assay

5-ethynyl-20-deoxyuridine (EdU) assay kit (Ribobio, Guangzhou, China), respectively, were used to measure cell proliferation according to the manufacturer's instructions. EdU incorporation assay was carried out according to previous studies [45, 46]. The experiments were performed in triplicate.

### Cell migration assay

Cell migration was observed by scratch assay following the reported methods [47]. Cells were seeded in 6-well plates and incubated in incubator to get 100% confluence before transfection. The cells were transfected with siRNAs or plasmids. A sterile 200 μl pipette tip was used to generate a clear line in the wells. Pictures were taken from each well quickly using a digital camera system. After one day, pictures were taken again. Migration distance was counted at the time of 0 h and 24 h. Experiments were carried out at least three times.

### Caspase-3 Elisa assay

Bladder cancer T24, 5637 and sw780 cells were transfected with siRNAs or plasmids in 12-well plates. After 48 hours, the activity of caspase-3 was measured by the caspase-3 enzyme-linked immunesorbent assay (ELISA) assay kit (Hcusabio, Wuhan, China) according to the manufacturer's instructions [45, 46]. Each test was performed at least three times.

### Hoechst 33258 staining assay

Bladder cancer T24, 5637 cells and SW780 were transfected with siRNAs or plasmids in 12-well plate. After 48 hours, the Hoechst 33258 staining kit (Life, Eugene, OR, USA) was employed to observe the apoptotic cells induced by siRNAs or plasmids [45, 46]. Assays were performed in triplicate.

### Flow cytometry analysis of cell apoptosis

Cells were cultivated in 6-well plates before transfection. Cells were transfected with siRNAs or plasmids. 2 days after transfection, cells were harvested and double stained with FITC-Annexin V and PI using the FITC Annexin V Apoptosis Detection Kit (TransGen, Perking, China) according to manufacturers’ protocol [45, 46]. Flow cytometry (EPICS, XL-4, Beckman, CA, USA) was used to detect cell apoptosis. In the pictures, cells were classified into dead cells, early apoptosis cells, living cells and late apoptotic cells. The ration of early apoptotic cells was considered as an observation index between negative control and experimental group. Each experiment was carried out in triplicate.

### Statistical analysis

All data were presented as mean ± standard deviation (SD) from three independent experiments. All statistical analyses were executed by using SPSS 21.0 software (IBM, Chicago, USA). The data of ABHD11-AS1 expression difference between bladder cancer tissues and adjacent normal tissues were analyzed using Paired samples’ *t* test. The data of CCK8 and MTT assays were analyzed by ANOVA. The data of cell migration and apoptosis assays were analyzed utilizing independent samples’ *t*-test. Differences were considered statistically significant according to p<0.05. Assays were performed in triplicate.
